# Pulse Oximetry with Two Infrared Wavelengths without Calibration in Extracted Arterial Blood

**DOI:** 10.3390/s18103457

**Published:** 2018-10-15

**Authors:** Ohad Yossef Hay, Meir Cohen, Itamar Nitzan, Yair Kasirer, Sarit Shahroor-karni, Yitzhak Yitzhaky, Shlomo Engelberg, Meir Nitzan

**Affiliations:** 1 Department of Physics/Electro-Optics Engineering, Jerusalem College of Technology, 21 Havaad Haleumi St., 91160 Jerusalem, Israel; ohadyh@gmail.com (O.Y.H.); mecohen@g.jct.ac.il (M.C.); 2 Department of Electro-Optical Engineering, Ben-Gurion University of the Negev. 1 Ben-Gurion Blvd, 8410501 Beer Sheva, Israel; ytshak@bgu.ac.il; 3 Department of Neonatology, Shaare Zedek Medical Center, Shmuel Bait St 12, 9103102 Jerusalem, Israel; itamarnitzan@gmail.com (I.N.); yairkasirer@yahoo.com (Y.K.); 4 Pediatric Intensive Care Unit, Shaare Zedek Medical Center, Shmuel Bait St 12, 9103102 Jerusalem, Israel; saritsk@hotmail.com; 5 Department of Electrical and Electronics Engineering, Jerusalem College of Technology, 21 Havaad Haleumi St., 91160 Jerusalem, Israel; shlomoe@jct.ac.il

**Keywords:** oxygen saturation, pulse oximetry, infrared, Beer–Lambert Law, calibration

## Abstract

Oxygen saturation in arterial blood (SaO_2_) provides information about the performance of the respiratory system. Non-invasive measurement of SaO_2_ by commercial pulse oximeters (SpO_2_) make use of photoplethysmographic pulses in the red and infrared regions and utilizes the different spectra of light absorption by oxygenated and de-oxygenated hemoglobin. Because light scattering and optical path-lengths differ between the two wavelengths, commercial pulse oximeters require empirical calibration which is based on SaO_2_ measurement in extracted arterial blood. They are still prone to error, because the path-lengths difference between the two wavelengths varies among different subjects. We have developed modified pulse oximetry, which makes use of two nearby infrared wavelengths that have relatively similar scattering constants and path-lengths and does not require an invasive calibration step. In measurements performed on adults during breath holding, the two-infrared pulse oximeter and a commercial pulse oximeter showed similar changes in SpO_2_. The two pulse oximeters showed similar accuracy when compared to SaO_2_ measurement in extracted arterial blood (the gold standard) performed in intensive care units on newborns and children with an arterial line. Errors in SpO_2_ because of variability in path-lengths difference between the two wavelengths are expected to be smaller in the two-infrared pulse oximeter.

## 1. Introduction

### Measurement of Oxygen Saturation in Arterial Blood

The primary mechanism of oxygen transport from the lungs to the cells of the body is via hemoglobin molecules in red blood cells. The oxygenation level of hemoglobin in arterial blood is estimated by the oxygen saturation in arterial blood, SaO_2_, which is defined as the ratio of the concentration of oxygenated hemoglobin to the concentration of total hemoglobin in the blood. SaO_2_ provides information about the performance of the respiratory system, and normal values of SaO_2_ are 94–98% for healthy adults at sea level.

**Figure 1:**
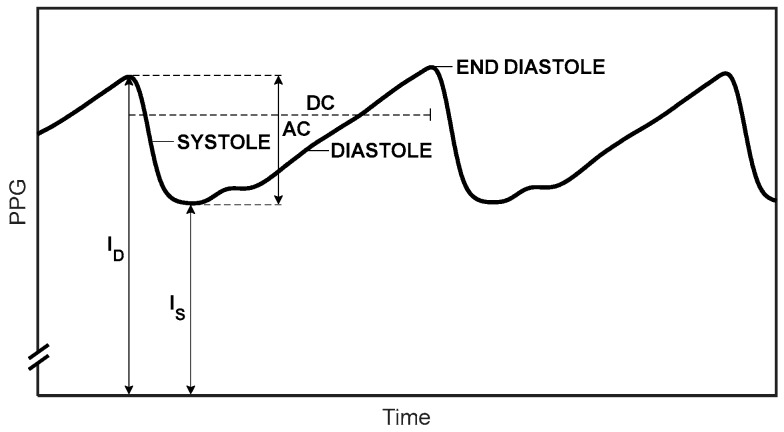
The photoplethysmography (PPG) pulses. The transmitted light through the tissue decreases during systole and increases during diastole. AC is the difference between the maximal (I_D_) and minimal (I_S_) light transmission through the tissue; DC is the mean light transmission through the pulse.

SaO_2_ can be assessed in extracted arterial blood by means of co-oximetry or by measurement of blood gases. SaO_2_ can also be obtained noninvasively in vivo by pulse oximetry, which utilizes the different light absorption spectra for oxygenated and deoxygenated hemoglobin. In order to isolate the contribution of the arterial blood to the light absorption, photoplethysmography (PPG)—the measurement of light absorption changes due to cardiac-induced arterial blood volume changes—is used. Light transmission through tissue decreases during systole because of the increase in the arterial blood volume during systole and increases during diastole (Figure 1): the PPG signal reflects light absorption changes due to arterial blood volume changes. Due to the discrepancy between the oxygen saturation values obtained by measurements in extracted blood and by in vivo pulse oximetry, the latter are denoted by SpO_2_.

Several studies have shown a potential error of 3–4% between SpO_2_ and direct SaO_2_ measurements in extracted blood in critically ill adult patients [1,2]. An even larger error is expected in neonates and children in intensive care units [3,4,5,6]. 

The theory of conventional pulse oximetry has been described in several publications [7,8,9,10], and some aspects of it, relevant to our study, will be presented here. A PPG-based parameter R—the ratio of ratios—is defined by

(1) $$R=\frac{{\left(AC/DC\right)}_{1}}{{\left(AC/DC\right)}_{2}}\;$$

where AC and DC are the peak-to-peak amplitude and the mean value of the PPG pulse, respectively (Figure 1), and the subscripts 1 and 2 refer to the two wavelengths. Utilizing the Beer–Lambert equation, the measured parameter R and the physiological parameter SaO_2_ are related through Equation (2) [7,8,9]:

(2) $$SaO_{2}=\frac{\varepsilon_{D1}-R\varepsilon_{D2}}{R(\varepsilon_{O2}-\varepsilon_{D2})+(\varepsilon_{D1}-\varepsilon_{O1})}\;$$

where $\varepsilon_{O}$ and $\varepsilon_{D}$ are the extinction coefficients for HbO_2_ and Hb, respectively. Equation (2) is valid if light attenuation in tissue is only due to absorption by hemoglobin and scattering effects can be neglected. In addition, the relationship between SaO_2_ and R, given in Equation (2), is valid when AC/DC<<1. Otherwise, R in Equation (2) should be replaced by R’, where R’ is defined as the ratio of $ln\left(I_{D}/I_{S}\right)$ for the two wavelengths:

R’ = [ln(I_D_/I_S_)]_1_/[ln(I_D_/I_S_)]_2_ (3)

The accuracy of Equation (2) is also limited because of the limited precision with which the values of the extinction coefficients of HbO_2_ and Hb are known; different studies provided significantly different data [11]. Since light scattering also contributes to light attenuation by increasing the path-lengths of the photons, and since the scattering constant and consequently the path-lengths increase depends on the wavelength, a correction term needs to be added to Equation (2) [9]:

(4) $$SpO_{2}=\frac{\varepsilon_{D1}-R(l_{2}/l_{1})\varepsilon_{D2}}{R(l_{2}/l_{1}){(\varepsilon }_{O2}-\varepsilon_{D2}{)+(\varepsilon }_{D1}-\varepsilon_{O1})}\;$$

where $l_{1}$ and $l_{2}$ are the path-lengths for the two wavelengths. The path-lengths ratio, $l_{2}/l_{1}$, for a given pair of wavelengths is not known when a specific examination is performed, and only a rough estimate of its mean value can be obtained from the literature (see [9]). Since $l_{2}/l_{1}$ varies between different subjects and between different clinical situations, assessment of SpO_2_ from R through Equation (4) is subject to error due to $l_{2}/l_{1}$ variability, which increases as the difference between $l_{2}$ and $l_{1}$ increases.

As a consequence of the insufficient accuracy in the published data of both the path-lengths ratio and the hemoglobin extinction coefficients, the relationship between the clinical parameter SaO_2_ and the measured parameter R in pulse oximetry was found empirically for each type of commercial pulse oximeter sensor by calibration [7,12]. The calibration includes measurements of R in several healthy volunteers simultaneously with in vitro SaO_2_ measurement in extracted arterial blood by means of a co-oximeter, for several values of SaO_2_, obtained by reducing the oxygen fraction in the inhaled air. Utilizing the acquired set of R/SaO_2_ pairs, an empirical calibration formula such as:

(5) $${\mathrm{SpO}}_{2}=\frac{k_{1}-k_{2}R}{k_{3}-k_{4}R}\;$$

can be created [7], where $k_{1}-k_{4}$ are constants, determined from the experimental R/SaO_2_ pairs, for each pair of wavelengths. Equation (5) is based on Equation (4). Different empirical calibration formulae have also been used, as well as lookup tables [10,13,14].

As explained above, the assumption that there are constant factors $k_{1}-k_{4}$ relating R and SaO_2_ for a given pair of wavelengths leads to an inevitable error since the use of $k_{1}-k_{4}$ values provides a single mean value for the path-lengths ratio, $l_{2}/l_{1}$, while the latter is subject to variability between persons and clinical situations. The effect of those fluctuations on the accuracy of SaO_2_ measurement increases as the path-lengths $l_{1}$ and $l_{2}$ differ from one another. For wavelengths in the red and infrared regions (such as 660 nm and 940 nm in some commercial pulse oximeters), the difference in scattering constant is significant—about 15% [15].

By using two nearby wavelengths in the infrared region, the difference between the corresponding scattering constants and path-lengths is reduced [9,16]. In this case, the natural variation in the path-lengths ratio between persons is expected to lead to errors that are small relative to those experienced when using pulse oximetry that uses two wavelengths, one of which is in the red and the other in the infrared region. In our study in 2014 [16], measurements on healthy persons by a pulse oximeter which was based on a pair of laser diodes with wavelengths of 780 nm and 808 nm exhibited SpO_2_ values between 95.3% and 100.5%. These results were similar to those of a commercial pulse oximeter: the maximal difference between them for each examinee was 2.5%.

The pulse oximeter in that study has two major limitations: the use of laser diodes and the use of two wavelengths that are very close. Laser diodes are expensive, inconvenient to use and require temperature stabilization. The difference between the two wavelengths used in that study was very small, rendering the values of the corresponding extinction coefficients very close. As a result, the calculation of SpO_2_ from Equation (2) or Equation (4) became very sensitive to small errors in the measurement of the PPG pulse amplitude, which is the basis for R measurement. In the current study, we measured SpO_2_ by a pulse oximeter with a dual light emitting diode (LED) with two infrared wavelengths and compared the results with SpO_2_ measured by a commercial pulse oximeter, with two wavelengths in the red and infrared regions.

## 2. Materials and Methods

Approval of the study was given by the Shaare Zedek Medical Center Institutional Review Board on 27 July 2014 (#32/14) and was extended on 17 August 2015 and 26 July 2017. Informed consent was obtained from each adult who was examined and from the parents of each examined child and newborn.

### 2.1. The Pulse Oximeter

The pulse oximeter with a dual LED with two infrared wavelengths (761 nm and 818 nm) was designed and constructed at the Jerusalem College of Technology (JCT). The JCT pulse oximeter was compared to commercial Nellcor pulse oximeters that make use of two wavelengths in the red and infrared regions (665 nm and 894 nm). The JCT pulse oximeter consisted of an optical probe, an electronic device and a laptop computer, for displaying the PPG signals and analyzing them. 

**Figure 2:**
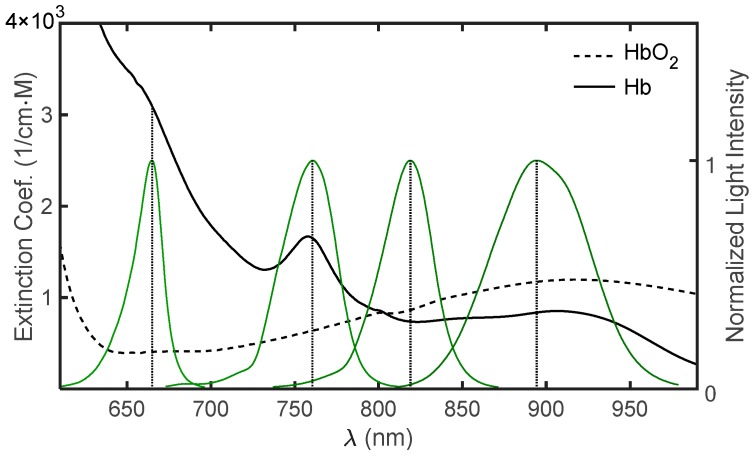
The normalized emitted light intensity spectra, obtained for light emitting diodes (LEDs) with peak wavelengths (λ) of 665 nm, 761 nm, 818 nm and 894 nm, and the extinction coefficient curves of Hb and HbO_2_, according to Zijlstra’s results.

The probe included a dual light emitting diode (LED), emitting light in two infrared wavelengths, and a photodetector. The emitted light spectra of the two infrared LEDs and the red and infrared LEDs of the Nellcor pulse oximeter probe (measured by a spectrometer, AvaSpec-2048, Avantes, The Netherlands) are presented in Figure 2, together with the extinction coefficient curves of Hb and HbO_2_, according to Zijlstra’s results [11,17].

**Figure 3:**
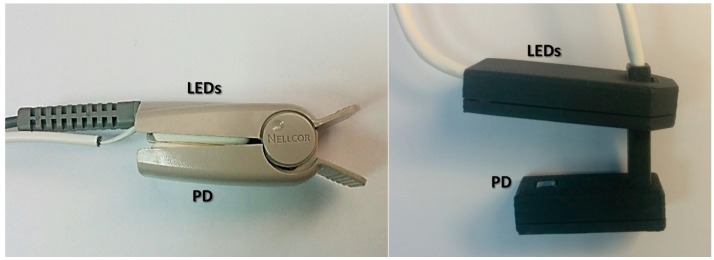
Photographs of the Jerusalem College of Technology (JCT) probes for use with an adult’s finger (**left**) and for use with a newborn’s foot or child’s finger (**right**). Light is emitted from one component of the probe and the light transmitted through finger/foot is collected by a detector that is placed on the other side of the finger/foot. PD—photodetector.

The finger probe for adults used with the JCT pulse oximeter consisted of a Nellcor finger probe with a soft contact surface, in which the Nellcor dual LED which emitted light with wavelengths of 665 nm and 894 nm was replaced by a dual LED emitting light at two infrared wavelengths. The foot probe for newborns was composed of two facing rigid plastic elements, with a small variable angle between them (Figure 3). The probe was constructed by a three-dimensional (3D) printer at JCT.

**Figure 4:**
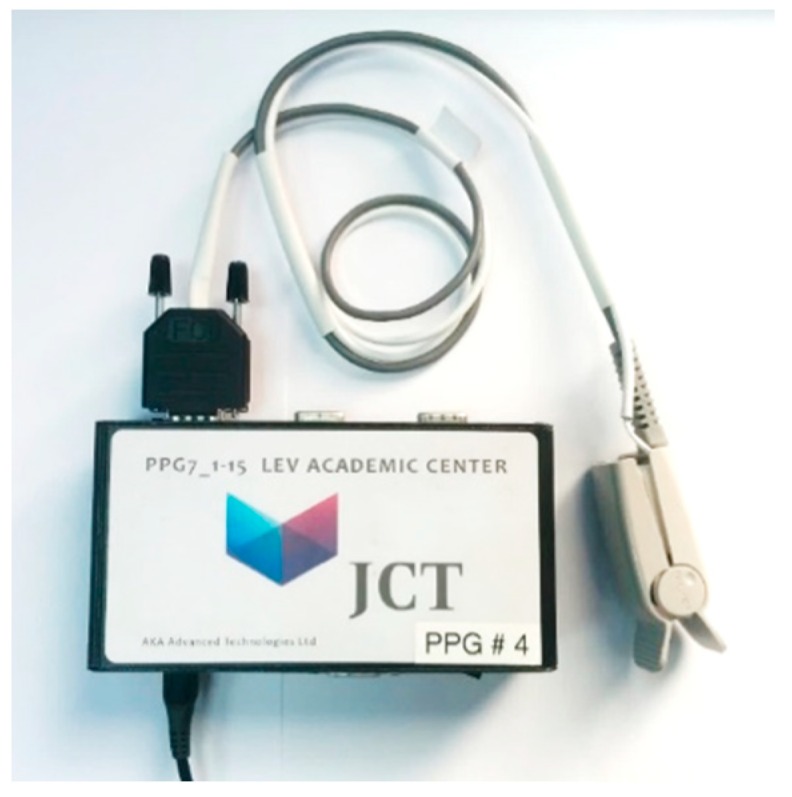
The control unit of the JCT pulse oximeter device together with an adult finger probe.

The control unit of the JCT pulse oximeter (Figure 4) controlled the current to the LEDs in the probe and digitized the photodetector output. Light from the two LEDs was emitted sequentially, by time-sharing the current supplied to the LEDs. The transmitted light of each wavelength was detected correspondingly by the photodetector, in synchronization with the current time-sharing. The signal was digitized (at 8000 samples/s with a 16-bit analog to digital converter) and every group of eight samples were averaged. The resultant signal, sampled at 1000 samples/s, was conveyed to a laptop for offline analysis using MATLAB. The power to the PPG device was supplied by batteries.

### 2.2. Determination of SpO_2_

SpO_2_ was determined by using a modified version of Equation (2):

(6) $$SpO_{2}=\frac{\varepsilon_{D1}-R\prime \varepsilon_{D2}}{R’(\varepsilon_{O2}-\varepsilon_{D2})+(\varepsilon_{D1}-\varepsilon_{O1})}\;$$

where R’ is as given in Equation (3). As explained above, Equation (2) (with R as defined in Equation (1)) is valid if $I_{D}-I_{S}$ is small relative to $I_{S}$. We have found that when $I_{D}-I_{S}$ is more than 3% of $I_{S}$, the difference between R and R’ cannot be neglected, and in this study, the ratio of ratios was calculated as the ratio of $ln\left(I_{D}/I_{S}\right)$ for the two wavelengths.

As described in our article in 2014 [16], the peak intensity of the PPG pulse, $I_{D}$, exhibits fluctuations of low and high frequencies, and the value of the PPG signal at the time of the pulse peak (end-diastole) might be different than what its value would have been at the time of the pulse minimum. The value of $I_{D}$, used for a given pulse, was taken as the value of the point of the line connecting the two maxima of the pulse at the time of the pulse minimum (see Figure 7 in [16]).

In order to derive SpO_2_ from Equation (6), the values of $\varepsilon_{O}$ and $\varepsilon_{D}$ must be known at the relevant wavelengths. As explained in the Introduction, the absorption spectra for Hb and HbO_2_ were measured by several research groups and their reported values differed from group to group [11]. Since the results of our previous study [16] were in best agreement with the extinction coefficient data of Zijlstra, we used those data in the current study.

In our study described in [16], we used laser diodes, which had narrow spectra. In the current study, we used LEDs, which had relatively broad spectra (Figure 2)—they had spectral widths of several tens of nanometers, so that the values of $\varepsilon_{O}$ and $\varepsilon_{D}$ changed along the spectrum. The mean extinction coefficients of Hb and HbO_2_ for the LED lights were taken as the weighted mean extinction coefficients, $\varepsilon_{O_{M}}$ and $\varepsilon_{D_{M}}$, defined by:

(7) $$\varepsilon_{D_{M}}=\frac{\sum_{i}\varepsilon_{D}\left(\lambda_{i}\right)\cdot I\left(\lambda_{i}\right)}{\sum_{i}I\left(\lambda_{i}\right)},\;\varepsilon_{O_{M}}=\frac{\sum_{i}\varepsilon_{O}\left(\lambda_{i}\right)\cdot I\left(\lambda_{i}\right)}{\sum_{i}I\left(\lambda_{i}\right)}\;$$

where $\varepsilon_{D}(\lambda_{i})$ and $\varepsilon_{O}(\lambda_{i})$ are the extinction coefficients at the wavelength $\lambda_{i}$ for Hb and HbO_2_, respectively, and $I(\lambda_{i})$ is the light intensity of the LED at $\lambda_{i}$. The variable i is an index that designates the wavelength in the LED spectrum. $I(\lambda_{i})$ for each LED was measured using a spectrometer and the values of Zijlstra $\varepsilon (\lambda_{i})$ were taken from the Kim and Liu articles [11,17]. For the wavelength range of 450–800 nm, Zijlstra’s data were given every two nanometers, and in the range of 800–1000 nm, data were only presented for nine wavelengths. The values of $\varepsilon_{D}(\lambda_{i})$ and $\varepsilon_{O}(\lambda_{i})$ were determined from Zijlstra’s data by using cubic spline interpolation.

### 2.3. Subjects and Methods

In the framework of the study, we compared SpO_2_ values obtained by the JCT pulse oximeter, which is based on two infrared wavelengths, with values obtained by a commercial pulse oximeter (Nellcor Bedside SpO₂, Covidien-Medtronic, Minneapolis, MI, USA), in measurements on 32 healthy male adults who were asked to hold their breath. After obtaining informed consent, the Nellcor probe, with one wavelength in the red and one in the infrared wavelengths, was attached to the examinee’s index finger and the JCT probe, with two infrared wavelengths, was attached to the examinee’s middle finger on the same hand. The examinee was asked to breathe according to a predetermined pattern that was displayed on a PC screen: regular breathings of 2.5 s inspiration and 2 s expiration for 30 s, then breath holding for 30 s, followed by regular breathings (the same as in the first stage) for 60 s. During the examination, the SpO_2_ values of the two pulse oximeters were recorded every 10 s, and the correlation between the SpO_2_ results of the two devices was calculated.

We also compared SpO_2_ measurements by the JCT pulse oximeter and a commercial pulse oximeter (Nellcor Bedside SpO_2_, Covidien-Medtronic, Minneapolis, MN, USA) with SaO_2_ measurement in extracted blood from an artery in a hospital setting. SaO_2_ measurement using extracted arterial blood and performed by a co-oximeter is the gold standard of SaO_2_ measurement. In the current study, non-invasive SpO_2_ was measured by the two pulse oximeters and was compared to in vitro SaO_2_ measurements in extracted blood from the umbilical artery in neonates or from an artery in the hand or foot in children. The examinations were performed in the neonatal intensive care unit (NICU) and in the pediatric intensive care unit (PICU), both at Shaare Zedek Medical Center, Jerusalem, Israel. The blood was extracted for medical purposes in order to monitor the patient’s blood gases. The SaO_2_ measurement in extracted blood was performed by means of a commercial co-oximeter (ABL90-Flex analyzer, Radiometer, Brønshøj, Copenhagen, Denmark). Informed consent was obtained from the parents of the children and the neonates.

The SpO_2_ measurements on the neonates were performed on the two feet of the examinee, using the JCT probe shown in Figure 3, which included a dual LED with two infrared wavelengths, 761 nm and 818 nm. The examinations on children were performed on the fingers using the same probe. The Nellcor probe consisted of a flexible band that was attached to the foot of the neonate in the NICU or to a finger of the child in the PICU.

## 3. Results

### 3.1. SpO_2_ Measurement in Breath Holding

In this study, SpO_2_ was measured on 32 healthy examinees who were asked to hold their breath for a period of 30 s (as described in the Materials and Methods Section). The SpO_2_ examinations were performed using a Nellcor pulse oximeter and the JCT pulse oximeter. The latter, which makes use of Equation (2), showed consistently lower SpO_2_ values than the former, probably due to the factor $l_{2}/l_{1}$ that should multiply R in Equation (4). Taking $\lambda_{1}$ as the lower wavelength, $l_{1}$ is greater than $l_{2}$ since the scattering constant decreases with increasing wavelength, and $l_{2}/l_{1}$ is smaller than one. When R in Equation (2) is replaced by $R(l_{2}/l_{1})$, SpO_2_ increases [9].

**Figure 5:**
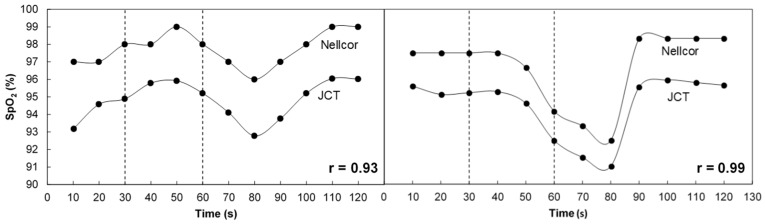
SpO_2_ values of two examinees as a function of time, as measured by the two pulse oximeters. The period of breath holding is demarcated by the two vertical lines.

Figure 5 presents the SpO_2_ values of two of the examinees during the desaturation and re-saturation periods, as measured by the two pulse oximeters. Note that the display of the Nellcor device shows the SpO_2_ results with a resolution of 1%, while the resolution of the JCT device was 0.1%. In the examination described in the left figure, SpO_2_ increased during the first 50 s of the examination, then decreased—effects that appeared in the majority of the examinations. In the other examination, SpO_2_ remained almost constant during the first 40 s of the examination, then decreased. In both examinations, SpO_2_ decreased until about 80 s due to the breath holding and then increased.

In general, the SpO_2_ vs. time curves for the different participants exhibited similar patterns. In the first 40–60 s of the examination, SpO_2_ either remained constant or increased by 1–4%. About 20–40 s after the subject started holding his breath, the majority of the participants showed a decrease in SpO_2_ of up to 5% for about 30 s, and then SpO_2_ increased. Note the delay in the decrease of SpO_2_ in the finger after the subject started holding his breath. Delays in the detection of hypoxemia when the pulse oximeter probe is placed on the finger have been reported [18,19].

**Figure 6:**
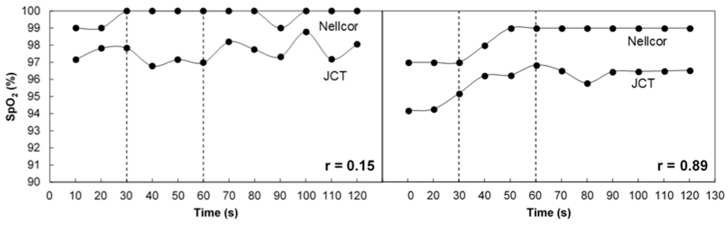
SpO_2_ values obtained by the Nellcor and JCT pulse oximeters in two examinations with a relatively low correlation coefficient.

The correlation between SpO_2_ measured by the two pulse oximeters in the individual examinations was high: in 30 out of 32 examinations, the correlation coefficient was higher than 0.50 (in two examinations the correlation coefficient was 0.155 and 0.45). The mean correlation coefficient for all the examinations was 0.86 and the median was 0.91, and in only eight examinations was the correlation coefficient lower than 0.85. In some examinations, the change in SpO_2_ due to breath holding was absent or small; in these examinations, even small discrepancies between the two pulse oximeters, such as those due to the rounding of the SpO_2_ values to whole digits in the commercial devices, could reduce the correlation between SpO_2_ values measured by the two pulse oximeters. Figure 6 presents two examinations with a relatively low correlation coefficient in which the change in SpO_2_ due to breath holding was absent or small. In the left figure, the SpO_2_ values obtained by the JCT pulse oximeter demonstrated various values between 96.3% and 98.3%, while the Nellcor SpO_2_ values only showed values of 99% and 100%.

For more reliable assessment of the correlation between the two devices, we only selected examinations in which SpO_2_ values decreased by 1.5% or more after breath holding. Such a criterion ensures that the Nellcor device, which only displays whole numbers, also shows the desaturation due to breath holding. Twenty-five examinations fulfilled this criterion, and the average correlation coefficient between SpO_2_ values obtained for this group by the two pulse oximeters was 0.91 ± 0.085. The correction factor for the JCT pulse oximeter due to the difference in path-lengths between the two wavelengths [9] was 1.035 for the whole group of examinations. The correction factor was taken as the ratio of the mean Nellcor–SpO_2_ and the mean JCT–SpO_2_ for the whole group of examinations.

**Figure 7:**
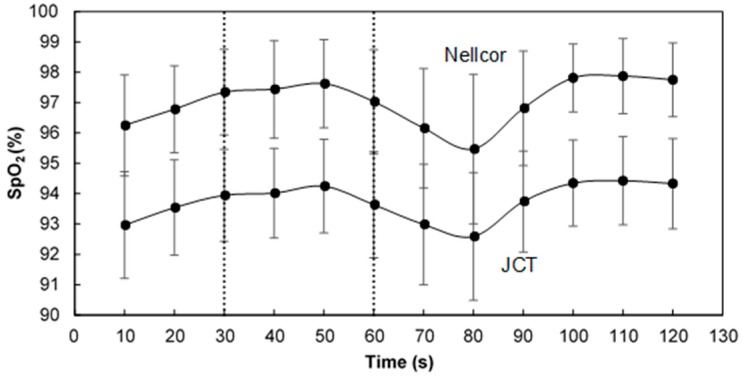
The mean SpO_2_ value (and standard deviation) as a function of time for the two devices, obtained for the whole group of examinees.

Figure 7 presents the mean SpO_2_ value as a function of time for the two devices, obtained for the whole group of examinees. The mean value of SpO_2_ increased up to 20 s after the start of breath holding, then decreased for 30 s, then increased.

The increase of SpO_2_ by about 1% after the start of the examination happened for the majority of the examinees and may be attributed to the change in the respiration pattern, from spontaneous respiration to the guided breathings of 2.5 s inspiration and 2 s expiration at the beginning of the examination. The delay of about 20 s in the SpO_2_ decrease after the start of the breath holding and in the SpO_2_ increase after the end of the breath holding is probably due to the transit time of the blood to the fingers [19].

### 3.2. Comparison of SpO_2_ with Invasive Measurement of SaO_2_

In order to assess the accuracy of SpO_2_ measurement by the JCT pulse oximeter, SaO_2_ and SpO_2_ were measured on neonates and children in the NICU and PICU who had arterial lines. SpO_2_ was measured simultaneously by the JCT pulse oximeter and a Nellcor pulse oximeter shortly after an arterial blood sample was extracted from the examinee for clinical purposes. Direct measurement of SaO_2_ in extracted arterial blood is the gold standard for arterial oxygen saturation measurement.

Table 1 presents SpO_2_ and SaO_2_ values as obtained by the two pulse oximeters and the invasive technique in the NICU and PICU. The derivation of SpO_2_ by the technique that is based on two infrared wavelengths (JCT–SpO_2_) was done by using the mean extinction coefficient, calculated over the emission spectra of the LEDs, as described in Section 2. Table 1 also presents the ratio SaO_2_/JCT–SpO_2_ for each examination. The mean of SaO_2_/JCT–SpO_2_, 1.055, was taken as a correction factor for JCT–SpO_2_ in order to compensate for the difference in the path-lengths between the two infrared wavelengths. The two right columns present the difference between Nellcor–SpO_2_ and SaO_2_ and between the corrected value of JCT–SpO_2_ and SaO_2_.

The mean of the difference between Nellcor–SpO_2_ and SaO_2_ was 3.84% ± 1.24 and between the corrected JCT–SpO_2_ and SaO_2_ was practically zero (due to the correction factor) and had a standard deviation of 3.35%. In one of the neonates’ examinations, the discrepancy between Nellcor–SpO_2_ and SaO_2_ was 17%: SaO_2_ was 70% and Nellcor–SpO_2_ was 87%. It is known that SpO_2_ measurements made when SaO_2_ is below 80% are subject to larger errors than those in which measurements are made when SaO_2_ is above 80% [6,20], but this very large error seems to be exceptional. If we delete this outlier, the mean and standard deviation of the difference between Nellcor–SpO_2_ and SaO_2_ becomes 2.33% ± 0.66.

The correlation coefficient between Nellcor–SpO_2_ and SaO_2_ was 0.776, and after removing the measurement with a 17% discrepancy, the correlation coefficient increased to 0.857. The correlation coefficient between JCT–SpO_2_ and SaO_2_ was 0.845.

The positive value of the mean difference between Nellcor–SpO_2_ and SaO_2_ is in accordance with the discrepancy in SpO_2_ measurements in neonates found in Ross et al. [4]. The standard deviation of the two pulse oximeters was above 3%, similar to the standard deviation reported for the SpO_2_ deviation from SaO_2_ in neonates [4,5].

**Figure 8:**
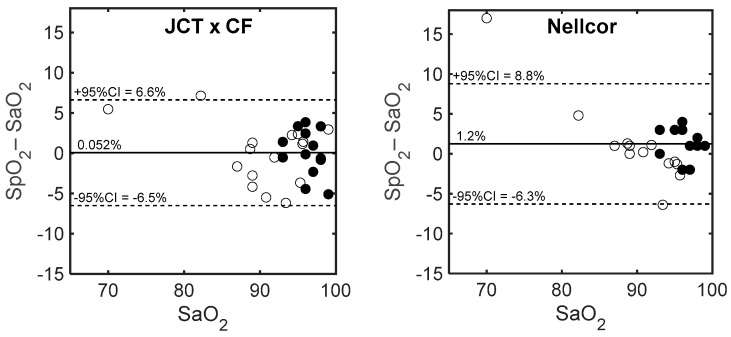
Bland–Altman plots of SpO_2_–SaO_2_ vs. SaO_2_ for Nellcor and JCT pulse oximeters are presented for the PICU children (full circles) and neonates (empty circles). The solid lines represent the mean of SpO_2_–SaO_2_ and the dashed lines are the 95% confidence interval (CI).

**Figure 9:**
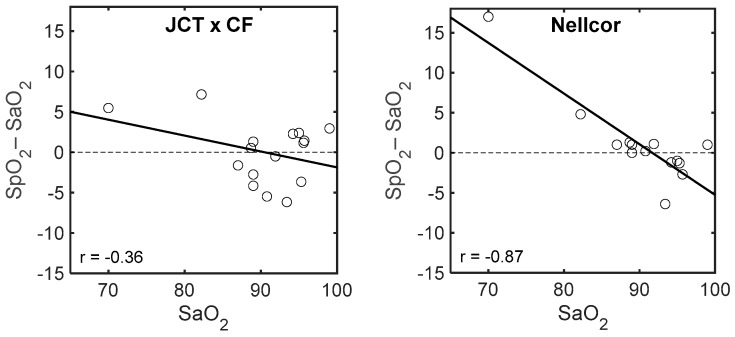
The regression lines of SpO_2_–SaO_2_ vs. SaO_2_ for Nellcor and JCT pulse oximeters for the neonate data.

Bland–Altman plots of SpO_2_–SaO_2_ vs. SaO_2_ for the Nellcor and the JCT pulse oximeters are presented in Figure 8. (In order to compare the parameter-difference for the two devices, the same parameter, SaO_2_, was used in the abscissa). The range of the SaO_2_ data of the PICU children was small: 93–100%, and that of the neonates was larger: 70–99%. The difference between JCT–SpO_2_ and SaO_2_ for the neonates was uniform through the SaO_2_ range, while for the Nellcor–SpO_2_ measurements, SpO_2_–SaO_2_ was positive for SaO_2_ below 85% and negative above 90%. The regression lines of SpO_2_–SaO_2_ vs. SaO_2_ for the Nellcor and JCT pulse oximeters for the neonate data are shown in Figure 9. The regression line for the JCT pulse oximeter is not statistically different from zero, while that for the Nellcor pulse oximeter differs significantly from zero (r = 0.87, *p* < 0.001).

## 4. Discussion

In the current study, we have developed a pulse oximeter which uses two wavelengths in the near infrared region and the relationship between the measured parameter, R, and the physiological parameter, SaO_2_, is based on the Beer–Lambert equation (Equation (2)). The technique requires the use of a correction factor to the calculated SpO_2_ value to account for the different path-lengths between the two wavelengths (Equation (4)). The R/SpO_2_ relationship in the available commercial pulse oximeters is based on the use of an empirical calibration formula (such as Equation (5)), which is obtained by means of in vitro measurements made on extracted arterial blood. The commercial devices use a dual LED with wavelengths in the red and infrared regions as a light source, in order to utilize the large difference in the extinction coefficient values between oxygenated and de-oxygenated hemoglobin in the red wavelength region (600–700 nm, see Figure 2). The large difference between the two wavelengths leads to a large difference between the corresponding scattering coefficients and consequently to a large difference between the red and infrared path-lengths. The large path-lengths difference is necessarily accompanied by large inter-person variability in the $l_{2}/l_{1}$ ratio, which might significantly affect the R/SpO_2_ relationship. The current JCT pulse oximeter used a dual LED that emits light at two relatively nearby infrared wavelengths. The effect of the small difference between the path-lengths of the two infrared wavelengths on the R/SpO_2_ relationship is small and can be compensated for by a correction factor that is not much different than unity. The reduced signal, caused by the smaller difference between the extinction coefficient values for Hb and HbO_2_, is compensated for by the reduction of the difference between the path-lengths for the two wavelengths, which are close to one another (i.e., reduced noise).

In a previous study [16], we used laser diodes, which have narrow bandwidth and distinct wavelengths, and showed that pulse oximetry with two nearby infrared wavelengths can provide oxygen saturation data without calibration which requires SaO_2_ measurements in extracted blood. In that study, the wavelengths of the laser diodes were very close to each other, 780 nm and 808 nm, so that the difference between the corresponding path-lengths was negligible, and no correction factor was needed. However, laser diodes are expensive, inconvenient to use and require temperature stabilization. Furthermore, the small distance between the two wavelengths is accompanied by a small difference between the extinction coefficients, which should lead to a small signal-to-noise ratio (see Equation (2)). In the current study, we used LEDs with broad emission spectra and wavelengths that were farther apart (761 nm and 818 nm). We showed that after applying a correction factor, the results were in good agreement with those of a Nellcor pulse oximeter.

The accuracy of the available commercial pulse oximeters is sufficient for many clinical applications, but not for all of them. In a study of critically ill patients [21], the authors concluded that changes in SpO_2_ do not reliably predict equivalent changes in SaO_2_ in critically ill patients. In two studies [1,2] on patients in intensive care units, a minimum SpO_2_ level of 94% or 96% has been proposed in order to ensure a minimal SaO_2_ value of 90%. Since excessive oxygen supplementation carries a risk of oxidative stress and oxygen toxicity [22,23], reduced error in SpO_2_ measurement has clinical significance. The limited ability of pulse oximetry to accurately detect excessive oxygenation is particularly important for preterm newborns receiving supplemental oxygen due to their vulnerability to retinopathy of prematurity when SaO_2_ levels are too high.

The greater sensitivity of preterm neonates to excessive oxygenation is particularly important because the error in SpO_2_ measurement in neonates and children in intensive care units is expected to be larger than that in adults [3,4,5,6]. One of the reasons for this larger error is limb movement by the neonates. While errors due to such movements can be reduced by signal processing algorithms, they cannot be totally eliminated. The movement error is not reduced by the JCT technique. Another source of error in newborns, particularly preterm neonates, is the presence of fetal hemoglobin. The relative differences in extinction coefficient values between adult hemoglobin and fetal hemoglobin for the wavelengths used in conventional pulse oximetry, 660 nm and 940 nm, are small [24], while in the 750–850 nm range, the relative differences seem to be more significant [24]. It should be noted that at sufficient time after birth or after infusion of blood with adult hemoglobin, the concentration of fetal hemoglobin is reduced and its effect on SpO_2_ measurement can be neglected. The calibration process, which is performed with extracted blood from healthy adults, is associated with another source of error. The relationship between SpO_2_ and R depends on the path-lengths ratio between the two wavelengths ($l_{2}/l_{1}$ in Equation (4)), and this ratio may be different in the fingers of adults, where the calibration is performed, and in the feet or hands of newborns, where the measurements are taken. As explained above, the variability of the path-lengths ratio between the two wavelengths should be larger when the difference between the two wavelengths is greater. 

The inaccuracy in SpO_2_ measurement by the available commercial pulse oximeters is increased for SaO_2_ levels below 80% [6,20]. In the calibration process, the oxygen fraction in the inspired air is not generally reduced below the safe value of 80% for SaO_2_, hence the lower effectivity of the calibration for SaO_2_ below 80%. In our study in the NICU, an exceptional error of 17% was obtained in SpO_2_ measurement by the Nellcor pulse oximeter in an examination where SaO_2_ was 70%, while the difference between JCT–SpO_2_ and the invasive SaO_2_ measurement was 1.5%.

## 5. Conclusions

The inaccuracy in the measurement of oxygen saturation in arterial blood by pulse oximetry is 3–4% for adults and greater for neonates. In part, the inaccuracy originates from the significant difference in the optical path-lengths between the red and infrared wavelengths used in the available pulse oximeters. In the current study, a novel pulse oximeter which used a dual LED with two nearby infrared wavelengths was introduced and examined. SpO_2_ was obtained from the measured parameter R through an equation which is based on the modified Beer–Lambert law and the extinction coefficient values for oxygenated and de-oxygenated hemoglobin. The two-infrared pulse oximetry does not require empirical calibration, which is based on SaO_2_ measurement in extracted arterial blood, as is required by red and infrared pulse oximetry. Despite the greater technological challenge in utilizing two nearby wavelengths, the two-infrared pulse oximeter and a commercial pulse oximeter showed similar changes in SpO_2_ in measurements performed on adults during breath holding. The two pulse oximeters showed similar accuracy when compared to SaO_2_ measurement in extracted arterial blood (the gold standard) performed in intensive care units on newborns and children with an arterial line. The accuracy of SpO_2_ measurement by the two-infrared pulse oximeter is expected to be greater than that of the red and infrared pulse oximeter because of smaller inter-person variability of the optical path-lengths difference between the two wavelengths.

## Figures and Tables

**Table 1:** SpO_2_ values as obtained by the Nellcor pulse oximeter and the JCT pulse oximeter and SaO_2_ measured by an invasive technique in the pediatric intensive care unit (PICU) (first 13 examinations) and the neonatal intensive care unit (NICU) (last 16 examinations). Also shown are SaO_2_/JCT–SpO_2_ and the difference (marked by Δ) between SaO_2_ and SpO_2_, as obtained by each pulse oximeter (with the correction, in the case of the JCT pulse oximeter). CF—correction factor.

Number	SaO_2_	SpO_2_		SaO_2_/SpO_2_ JCT	SpO_2_ JCT × CF	ΔSpO_2_ JCT	ΔSpO_2_ Nellcor
		Nellcor	JCT				
1	98	100	92.3	1.06	97.4	−0.63	2.00
2	98	99	92.1	1.06	97.2	−0.83	1.00
3	96	94	86.8	1.11	91.5	−4.45	−2.00
4	95	98	93.2	1.02	98.4	3.36	3.00
5	97	98	89.7	1.08	94.7	−2.32	1.00
6	96	99	94.6	1.01	99.8	3.85	3.00
7	96	100	93.3	1.03	98.5	2.45	4.00
8	97	95	92.8	1.04	98.0	0.95	−2.00
9	98	100	96.0	1.02	101.3	3.34	2.00
10	93	96	87.7	1.06	92.5	−0.52	3.00
11	93	93	89.5	1.04	94.4	1.40	0.00
12	99	100	89.0	1.11	93.9	−5.10	1.00
13	96	99	90.9	1.06	95.9	−0.13	3.00
1	89	90	80.4	1.11	84.8	−4.16	1.00
2	96	93	92.0	1.04	97.1	1.12	−3.00
3	95	94	92.3	1.03	97.4	2.39	−1.00
4	82	87	84.7	0.97	89.3	7.35	5.00
5	89	90	81.7	1.09	86.2	−2.76	1.00
6	99	100	96.6	1.02	101.9	2.94	1.00
7	91	91	80.9	1.13	85.3	−5.68	0.00
8	93	87	82.7	1.12	87.2	−5.78	−6.00
9	96		91.7	1.05	96.8	0.76	
10	70	87	71.5	0.98	75.5	5.46	17.00
11	94	93	91.4	1.03	96.5	2.47	−1.00
12	92	93	86.6	1.06	91.4	−0.62	1.00
13	89	90	84.6	1.05	89.2	0.22	1.00
14	89	89	85.6	1.04	90.3	1.30	0.00
15	95	94	86.8	1.09	91.6	−3.36	−1.00
16	87	88	80.9	1.08	85.4	−1.64	1.00
Mean	93.0	94.2	88.2	1.055	93.1	0.05	1.25
Std	6.0	4.6	5.7	0.04	6.0	3.34	3.83
